# Association between geriatric 8 (G8) scores and self-care decline in elderly patients with head and neck squamous cell carcinoma undergoing radiotherapy

**DOI:** 10.1007/s00520-025-09936-2

**Published:** 2025-09-16

**Authors:** Tsuyoshi Katsuta, Ikuno Nishibuchi, Miki Fujioka, Megumi Nomura, Miho Kondo, Takao Hamamoto, Tsutomu Ueda, Hiroki Ochi, Hiroshi Sakauchi, Shigeyuki Tani, Nobuki Imano, Yuji Murakami

**Affiliations:** 1 Department of Radiation Oncology, Graduate School of Biomedical and Health Sciences, Hiroshima University, 1-2-3 Kasumi, Minami-Ku, Hiroshima, 734-8551 Japan; 2 Department of Nursing, Hiroshima University Hospital, 1-2-3 Kasumi, Minami-Ku, Hiroshima, 734-8551 Japan; 3 Department of Otorhinolaryngology, Head and Neck Surgery, Graduate School of Biomedical and Health Sciences, Hiroshima University, 1-2-3 Kasumi, Minami-Ku, Hiroshima, 734-8551 Japan; 4 Department of Radiation Oncology, Hiroshima Red Cross Hospital & Atomic-Bomb Survivors Hospital, 1-9-6 Sendamachi, Naka-Ku, Hiroshima City, Hiroshima, Japan

**Keywords:** Geriatric assessment, G8 screening tool, Elderly patients, Self-care, Head and neck cancer, Chemoradiotherapy

## Abstract

**Purpose:**

Self-care during radiotherapy (RT) is crucial for managing mucositis and dermatitis in patients with head and neck squamous cell carcinomas (HNSCC). However, elderly patients often struggle with self-care. This study examined the relationship between self-care decline and the Geriatric 8 (G8) score.

**Methods:**

A retrospective analysis was conducted on 66 patients (≥ 65 years) with HNSCC who met the inclusion and exclusion criteria and received definitive RT between December 2018 and February 2023. Self-care activities—medication adherence, oral care, grooming, skin ointment application, and gauze dressing—were assessed during definitive RT on a 0–5 scale. Patients were first grouped by initial self-care independence, and their G8 scores were compared. Among initially independent patients, those with self-care score changes were further analyzed based on their G8 scores. A threshold value was also determined to differentiate between the groups.

**Results:**

The initially independent group exhibited significantly higher G8 scores than non-independent group (median G8 score: 14 vs. 9.75, *P* = 0.0067). Among the initially independent patients, 15 (24.2%) experienced self-care decline, and lower baseline G8 scores significantly predicted this deterioration (median　G8 score: 12 vs. 15; *P* = 0.011). Multivariate analysis identified G8 scores as a significant predictor of self-care decline (odds ratio = 6.53, *P* = 0.0074). Receiver operating characteristic (ROC) analysis determined a G8 cutoff of 12 (area under the curve = 0.72) with 53.3% sensitivity and 85.1% specificity.

**Conclusion:**

These findings indicate a possible coherence between the absence of frailty and maintenance of self-care in elderly patients undergoing RT. Further, prospectively designed research is needed to confirm these findings in a larger cohort.

**Supplementary Information:**

The online version contains supplementary material available at 10.1007/s00520-025-09936-2.

## Introduction

Radiotherapy (RT) and chemoradiotherapy (CRT) are standard treatments for head and neck squamous cell carcinomas (HNSCC) [1–3]. These approaches are generally considered organ-preserving and allow for the maintenance of anatomical structures and essential functions, although adverse effects such as dysphagia may occasionally occur. RT often causes toxic effects like dermatitis and mucositis [4, 5], which can result in significant discomfort, pain, systemic infections, or treatment interruptions. Effective management of these side effects is essential to ensure treatment adherence and completion.

Treatment-related toxicities depend on factors such as treatment intensity, irradiated area extent, nutritional status, and supportive self-care [6–8]. Among these factors, self-care activities during treatment have gained increasing attention in recent years. For acute dermatitis, proper skin cleansing and prophylactic moisturizers promote recovery, and surgical pads can accelerate healing [8, 9]. To prevent mucositis progression, the Multinational Association of Supportive Care in Cancer (MASCC) and the International Society of Oral Oncology (ISOO) guidelines recommend the implementation of basic oral care [10].

Elderly patients face challenges maintaining self-care due to cognitive and physical decline [11]. Even within the same age group, health and functional abilities vary greatly, a phenomenon referred to as the “heterogeneity of the elderly,” which underscores the need for individualized care [12]. The American Society of Clinical Oncology (ASCO) and International Society of Geriatric Oncology (SIOG) recommend geriatric assessment (GA) as a gold standard to evaluate elderly patients’ health, predicting function, institutionalization risk, and mortality [13, 14]. However, since the implementation of GA requires significant human and time resources, several simplified GA screening tools have been developed [15, 16]. Among these tools, the geriatric-8 (G8) screening tool has been reported to be simple yet useful in identifying vulnerable patients [17].

Despite growing interest in self-care during RT, few studies have examined the relationship between self-care activities and GA among elderly patients with HNSCC. This study aimed to investigate the relationship between changes in self-care activities during definitive RT or CRT and G8 screening tool scores.

## Materials and methods

### Study design ethics and data collection

This retrospective study aimed to evaluate the relationship between changes in self-care activities during definitive RT or CRT and the G8 screening tool scores in patients aged 65 years or older with HNSCC treated at our institution between December 2018 and February 2023. The study was approved by the Certified Clinical Research Committee of Hiroshima University (Certification Number: E2019-1656–10) and conducted in accordance with the principles of the Declaration of Helsinki.

At our institution, the G8 screening tool has been routinely implemented in clinical practice for elderly patients undergoing RT since 2018. Furthermore, the assessment and nursing support of self-care activities—such as medication adherence, oral care, grooming, and skin care—have been part of routine clinical care even before that time. These evaluations were conducted by trained oncology nurses and documented in the electronic medical record (EMR) system. For this study, G8 scores and self-care assessment data were retrospectively extracted from the EMRs.

Because this study utilized anonymized data derived from EMR and involved no interventions or invasive procedures beyond routine clinical practice, individual informed consent was not required under institutional guidelines. In accordance with ethical regulations, information regarding the study and the opt-out procedure was made publicly available on the official website of our institution.

### Patients selection

Between 2018 and 2023, a total of 169 patients aged ≥ 65 years who underwent radiotherapy for head and neck cancer at our department were screened for eligibility. The tumor stage was classified according to the 8th edition of the American Joint Committee on Cancer Staging Manual and Handbook [18]. Inclusion criteria included: (1) definitive RT or CRT for HNSCC, (2) a prescribed dose of 60–70 Gy to the laryngopharyngeal region, and (3) age ≥ 65 years at the time of consent. Exclusion criteria were as follows: (1) age < 65 years (N = 77), (2) head and neck cancers other than nasopharyngeal, oropharyngeal, hypopharyngeal, laryngeal, or oral cancer (N = 13), (3) histological types other than squamous cell carcinoma (N = 1), (4) prior radiation therapy in the head and neck region (N = 0), (5) palliative RT (N = 5), and (6) patients deemed inappropriate by the principal investigator or research coordinator (N = 7), including 3 cases of superficial pharyngeal or early laryngeal cancer treated with localized RT only, and 4 cases in which RT was discontinued before reaching 60 Gy due to disease progression.

**Fig. 1:**
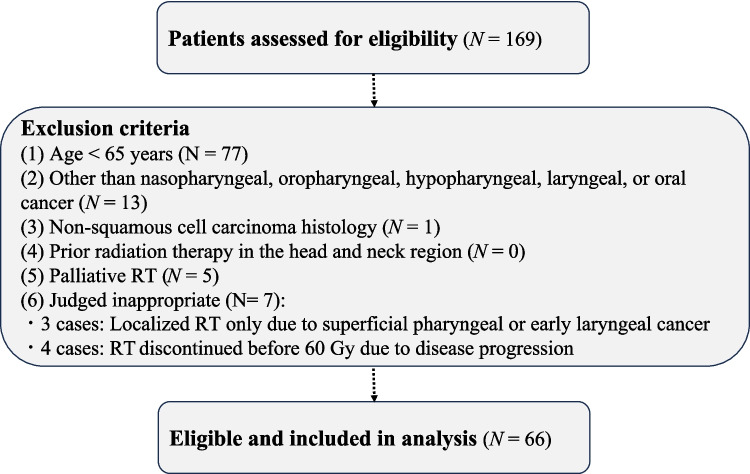
CONSORT flow diagram of patient selection. Abbreviations, CONSORT: Consolidated Standards of Reporting Trials. RT: radiation therapy

After applying these criteria, 66 patients were included in the final analysis. The patient selection process is illustrated in the CONSORT flow diagram (Fig. 1).

### Radiotherapy

Patients were immobilized using thermoplastic masks, and contrast-enhanced CT scans were obtained for treatment planning (Varian Medical Systems, Palo Alto, CA, USA). Treatment plans utilized volumetric modulated arc therapy (VMAT) with the simultaneous integrated boost technique. A total dose of 70 Gy in 35 fractions was administered to primary tumors and metastatic lymph nodes, 63 Gy to areas adjacent to the primary tumor, and 56 Gy to selective lymph nodes. For patients with widespread lymph node metastases, early-stage cancer, or advanced age, the simultaneous integrated boost method was replaced with a 2-step VMAT approach or reduced irradiated fields limited to the primary tumor and surrounding area. The treatment plan was delivered to the patient through TrueBeam linear accelerators (Varian Medical Systems).

### Chemotherapy

For fit patients with advanced or locally aggressive HNSCC, neoadjuvant chemotherapy was administered to reduce tumor volume [19]. The TPF regimen included docetaxel (70 mg/m^2^) on day 1, cisplatin (CDDP) (70 mg/m^2^) on day 1, and 5-fluorouracil (750 mg/m^2^) as a continuous 5-day infusion. Two cycles were delivered at 3–4-week intervals. Concurrent chemotherapy involved three cycles of CDDP (100 mg/m^2^) every three weeks. For patients unable to tolerate CDDP, carboplatin or cetuximab [20] was substituted. RT alone was performed for patients unsuitable for chemotherapy.

### Geriatric assessment and grading of the self-care activities

The G8 consists of eight items that assess various aspects of health and functional status: 1) food intake in the last 3 months, 2) recent weight loss (< 3 months), 3) mobility, 4) neuropsychological problems, 5) body mass index (BMI), 6) polypharmacy (take ≥ 3 medications per day), 7) self-perceived health status compared to the same age people, and 8) age. The total score ranges from 0 to 17 (Supplementary table S1), with lower scores indicating poorer general health status and frailty.

During therapy, patients were evaluated by radiation oncologists and radiation oncology nurses every 10 Gy to assess the severity of acute oral/pharyngeal radiation mucositis and neck radiation dermatitis based on the National Cancer Institute Common Terminology Criteria for Adverse Events (version 5.0). Simultaneously, face-to-face educational interviews, lasting approximately 20–30 min, were conducted by nurses. These interviews aimed to assess self-care activities related to managing radiation-induced side effects and to provide supportive care to promote patient actions. The following five activities were evaluated: 1) medication adherence, 2) oral care, 3) grooming, 4) skin ointment application, and 5) gauze dressing. For each self-care activity, one point was deducted if nursing intervention was required, resulting in a total point ranging from 0 to 5 (maximum: 5 points, minimum: 0 points). Specific criteria for judging self-care activities, such as whether or not needing the intervention, were based on the report of Nakano et al. [21].

Patients were initially classified into two groups based on their self-care independence at the beginning of treatment: those who were independent in self-care (initially independent group) and those who required nursing intervention (initially non-independent group). The difference in G8 scores between these two groups was analyzed to explore the relationship between baseline self-care abilities and frailty.

Further categorization was performed within the initially independent group, where patients were divided into those whose self-care abilities remained stable throughout treatment and those who eventually required nursing assistance. Differences in G8 scores between these subgroups were also examined. Additionally, among the five self-care activities, the most challenging one to maintain during treatment was identified.

### Statistical analysis

To evaluate the association between G8 scores and self-care independence, statistical analyses were conducted using Excel statistical software ver. 4.08 (BellCurve for Excel; Social Survey Research Information Co., Ltd., Tokyo, Japan). Statistical significance was set at *P* < 0.05.

Comparisons of G8 scores between the initially independent and non-independent group were performed using the Mann–Whitney U test. Similarly, comparisons of G8 scores between subgroups within the initially independent group—those who maintained independence versus those who required nursing assistance—were also conducted using the Mann–Whitney U test.

Additionally, logistic regression analysis was performed to explore potential predictors of self-care decline among clinical characteristics, excluding age, as it was already accounted for in G8 score. Variables with *P* < 0.1 in the univariate analysis were considered for the multivariate model to minimize overfitting, given the limited number of events. Based on this criterion, G8 score and Eastern Cooperative Oncology Group (ECOG) Performance Status (PS) were included in the final model. Fisher’s exact test was used to examine associations between categorical baseline characteristics and self-care decline.

To further evaluate significant findings, receiver operating characteristic (ROC) curve analysis was conducted to determine cutoff values, the area under the curve (AUC), sensitivity, and specificity, providing further insight into self-care deterioration in elderly patients undergoing RT.

## Results

From December 2018 and February 2023, 66 patients were enrolled. Their clinical data are summarized in Table 1. Sixty patients (90.9%) were male, and the mean age was 74.2 years (range: 67–94). ECOG PS was distributed as follows: 52 patients (78.8%) with PS 0, 12 patients (18.2%) with PS 1, and 2 patients (3.0%) with PS 2. The mean G8 score was 13.5 (range: 7–17), with a median score of 14. Details of G8 scores are provided in Supplementary table S2. Treatment settings included 63 patients (95.5%) treated as inpatients and 3 patients (4.5%) as outpatients.

**Table 1:** Patients’ and treatment characteristics of head and neck squamous cell carcinoma

Characteristic	Number of patients (*N* = 66)	( %)
Gender		
Male	60	90.9
Female	6	9.1
Age(years)		
Mean	74.2	
Range	67–94	
Performance status		
0	52	78.8
1	12	18.2
2	2	3.0
G8 score		
Mean	13.5	
Range	7–17	
Treatment setting		
Inpatient	63	95.5
Outpatient	3	4.5
Primary tumor site		
Nasopharynx	5	7.6
Oropharynx	15	22.7
Hypopharynx	33	50.0
Oral cavity and mouth floor	8	12.1
Larynx	5	7.6
Clinical stage		
Stage 0- I	5	7.6
Stage II	15	22.7
Stage III	15	22.7
Stage IVA and IVB	31	47.0
Total Dose (Gy) /fr		
70 Gy/35fr	63	95.5
66–68 Gy/33-34fr	2	3.0
62 Gy/31fr	1	1.5
Combination Chemotherapy^*^		
Cisplatin	45	68.2
Carboplatin	2	3.0
Cetuximab	9	13.6
RT alone	10	15.2
Self-care independence		
Independent	62	93.9
Need assistance	4	6.1

*; Concurrent chemotherapy only; neoadjuvant chemotherapy not included. *Abbreviations,* G8: geriatric 8, fr: fractions, RT: radiation therapy

The primary tumor sites included the nasopharynx (5 patients, 7.6%), oropharynx (15 patients, 22.7%), hypopharynx (33 patients, 50.0%), oral cavity and mouth floor (8 patients, 12.1%), and larynx (5 patients, 7.6%). Among oropharyngeal cases, 9 patients were tested for p16 expression. Disease stages were distributed as follows: Stage 0–I in 5 patients (7.6%), Stage II in 15 (22.7%), Stage III in 15 (22.7%), and Stage IVA–IVB in 31 (47.0%).

For radiotherapy, 63 patients (95.5%) received 70 Gy in 35 fractions, 2 patients (3.0%) received 66–68 Gy in 33–34 fractions, and 1 patient (1.5%) received 62 Gy in 31 fractions, with treatment discontinued due to chemotherapy complications. VMAT was used in 64 patients (97.0%), while 2 (3.0%) received three-dimensional conformal radiation therapy. Whole-neck irradiation, including prophylactic lymph node areas, was performed in 57 patients (86.4%), while 9 (13.6%) received a shrinking field technique. Neoadjuvant chemotherapy was given to 23 patients (34.8%) with TPF. Concurrent chemotherapy included CDDP in 45 patients (68.2%), CBCDA in 2 (3.0%), cetuximab in 9 (13.6%), while 10 patients (15.2%) received radiotherapy alone.

 During treatment, grade 3 mucositis and dermatitis were observed in 14 (21.2%) and 12 (18.1%) patients, respectively, with no grade 4 or higher adverse events.

### Comparison of G8 scores between initially independent and non-independent groups

At the start of treatment, 62 patients (93.9%) were independent in self-care (initially independent group), while 4 patients (6.1%) required nursing assistance (initially non-independent group). The initially independent group had a significantly higher baseline G8 score compared to the non-independent group (median G8 score: 14 vs. 9.75; *P* = 0.0067). This suggests that patients requiring nursing intervention at baseline were more frail and had poorer general health.

### Self-care maintenance during treatment

Within the initially independent group, 47 patients (75.8%) maintained their self-care independence throughout the treatment period, while 15 patients (24.2%) eventually required nursing intervention. The G8 score at baseline was significantly lower in the subgroup that experienced self-care decline compared to those who remained independent (median　G8 score: 12 vs. 15; *P* = 0.011). Declines of 1, 2, 3, and 4 points were observed in 3, 8, 2, and 2 patients, respectively. Skin ointment application was the most challenging activity, requiring assistance in 11 patients. Details of other items are listed in Table 2.

**Table 2:** Self-care activities for which nursing assistance was required at the end of RT

Self-care activities	Medication adherence	Oral care	Grooming	Skin ointment application	Gauze dressing
Number of patients (*N* = 15)	2	5	5	11	10

*Abbreviations,* RT: radiation therapy

### Predictors of self-care decline

Among clinical variables, G8 scores were significant predictors of self-care decline, while PS (*P* = 0.081) was not statistically significant but showed a trend toward significance in the univariate analysis (Table 3). In multivariate analysis, both variables reached significance, but G8 scores (OR = 6.53, 95% CI: 1.79–23.81, *P* = 0.0074) showed stronger predictive power compared to PS (OR = 3.42, 95% CI: 0.87–13.49, *P* = 0.029) (Table 4). To further examine the predictive performance of G8 and PS independently of age effects, we conducted an age-stratified ROC analysis. Patients were divided into two groups: 65–73 years and ≥ 74 years. In both age groups, the G8 score demonstrated higher AUC values compared to PS, with AUCs of 0.68 and 0.71 for G8 in the younger and older groups, respectively (Supplementary Table S3). These results suggest that the G8 score maintains superior predictive ability across age strata, supporting its utility beyond the age component it includes.

**Table 3:** Results of Univariate analysis

Variables	Self-care decline *N* = 15 (%)	Not self-care decline *N* = 47 (%)	*P*-value
Gender			
Male	14 (93.3)	44 (93.6)	0.68
Female	1 (6.7)	3 (6.4)	
Performance status			
0	10 (66.7)	41 (87.2)	0.081
1	5 (33.3)	6 (12.8)	
G8 score			
Median	12	15	**0.011**
Range	8–17	7–17	
Treatment setting			
Inpatient	15 (100)	44 (93.6)	0.43
Outpatient	0 (0)	3 (6.4)	
Clinical stage			
Stage 0- II	3 (20.0)	16 (34.0)	0.24
Stage III-Ⅳ	12 (80.0)	31 (66.0)	
Chemotherapy			
Yes	12 (80.0)	43 (91.5)	0.22
No	3 (20.0)	4 (8.5)	

*Abbreviations,* G8: geriatric 8. Bold type indicates that the *P* -value is equal to or less than the level of statistical significance (0.05)

**Table 4:** Multivariate logistic regression model for self-care decline

Variables	Odds ratio	95% CI	*P*-value
Performance status	3.42	0.87–13.49	**0.029**
G8 score	6.53	1.79–23.81	**0.0074**

*Abbreviations,* CI: confidence interval, G8: geriatric 8. Bold type indicates that the *P* -value is equal to or less than the level of statistical significance (0.05)

**Fig. 2:**
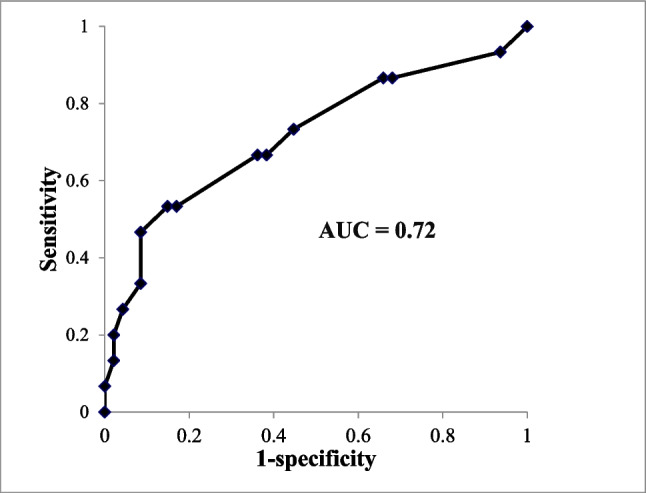
The receiver operator characteristic curve for Geriatric8 scores in the prediction of self-care activity decline. *Abbreviations*, AUC: area under the curve

Based on the overall cohort, ROC analysis identified a G8 score cutoff of 12 using the Youden Index, with an AUC of 0.72, sensitivity of 53.3%, and specificity of 85.1% (Fig. 2).

## Discussion

To our knowledge, this is the first study to quantitatively evaluate the association between G8 screening scores and self-care capacity in elderly patients undergoing definitive RT or CRT for HNSCC. The results demonstrate that lower G8 scores are significantly associated with a decline in self-care abilities during treatment. Patients who were initially independent in self-care but later required nursing assistance had significantly lower baseline G8 scores than those who maintained their independence. Additionally, a G8 score cutoff of 12 was identified as a predictor of self-care deterioration, with an odds ratio of 6.53.

GA had been widely recognized as essential tools for evaluating the overall health status of elderly patients undergoing cancer treatment. The G8 screening tool has been previously associated with treatment tolerance, chemotherapy-related toxicities, and survival outcomes in elderly cancer patients [22–26]. Our study adds to this growing body of evidence by demonstrating its predictive value in self-care maintenance during RT. The strong association between lower G8 scores and self-care decline suggests that frailty, as measured by G8, affects not only treatment outcomes but also the ability of elderly patients to manage side effects effectively. This study emphasizes the importance of early assessment and intervention to support self-care maintenance. The G8 screening tool demonstrated high specificity (85.1%), making it valuable for minimizing false positives among patients predicted to struggle with self-care. This specificity is particularly relevant in resource-limited settings, such as facilities with fewer nurses who need to care for a large number of patients undergoing treatment. By focusing care on at-risk patients identified through the G8, more efficient and effective supportive interventions can be implemented.

There are limited studies examining GA in the context of RT. Regarding RT-related toxicity, Cuccia et al. reported that lower G8 scores were associated with an increased likelihood of late toxicity in patients undergoing stereotactic radiotherapy for lung cancer [27]. Additionally, studies on instrumental activities of daily living have shown that lower G8 scores correlate with decreased functional independence [28]. Our findings align with these reports, further emphasizing the importance of assessing frailty in elderly cancer patients. However, unlike previous studies, our research specifically identifies self-care maintenance as a crucial factor influenced by baseline G8 scores in patients with HNSCC undergoing RT.

The ECOG PS, a commonly used functional measure in oncology, showed an association with self-care decline; while, the G8 tool demonstrated a stronger and more significant correlation. This highlights the limitations of traditional metrics in geriatric populations and underscores the G8 tool’s ability to capture broader health dimensions, including physical, cognitive, and nutritional status. These results align with previous findings suggesting the G8 is a more sensitive and comprehensive predictor of general condition in geriatric care [25, 29].

In this study, a G8 score of 12 was identified as the threshold. In other reports on the G8 screening tool, a score of ≤ 14 out of 17 points is considered abnormal [26, 30]. In patients with HNSCC, the progression of the disease　itself is closely linked to nutritional status, which may result in generally lower scores. Additionally, in this study, G8 scores were obtained at the time of referral to the radiation oncology department. Approximately 35% of the patients had already undergone neoadjuvant chemotherapy, and it cannot be ruled out that the deterioration of nutritional status caused by chemotherapy or the increased use of medications to manage chemotherapy-related side effects may have influenced their scores. Although the sensitivity of the cut-off value of 12 was limited (53.3%), the specificity was relatively high (85.1%), which aligns with our objective of identifying patients who are likely to maintain self-care during RT without requiring intensive nursing intervention. The cutoff value of 12 was selected based on the Youden Index, representing the best overall balance between sensitivity and specificity in this cohort. Nevertheless, further investigations are needed to validate whether similar results can be replicated in larger patient cohorts and in other cancer types. Furthermore, the potential impact of concurrent chemotherapy on self-care independence was not specifically assessed by the G8 screening tool. Given that concurrent chemotherapy may independently cause side effects such as fatigue, nausea, and general weakness, its influence on patients' ability to maintain self-care activities during RT should be explicitly evaluated in future studies.

We selected 5 commonly recommended self-care activities for managing radiation-induced dermatitis and mucositis with reference to previous research and clinical guidelines [7–10] in this study. For the assessment criteria, we adopted the framework proposed by Nakano et al. [21], which discusses standards for evaluating patients’ self-care levels in managing cancer-related symptoms. Currently, there is no standardized method for assessing self-care activities or their measurement specific to HNSCC field. Instrument such as the Exercise of Self-Care Agency Scale (ESCA) [31] is well-known but was developed for broader applications, including chronic illnesses, and not specific to cancer care. Furthermore, its large number of items makes them impractical for routine clinical use. The evaluation items and criteria employed in this study will require further refinement. However, given that they were designed to be specific to RT for HNSCC, simple, and as clearly defined as possible, we believe they are valid and broadly applicable in clinical practice.

Among the self-care activities, skin ointment application emerged as the most challenging, requiring nursing assistance in 11 patients. Reasons for the difficulty in proper ointment application included the anatomically complex structure of the head and neck region, as well as areas like the nape of the neck being hard to reach. These factors made it challenging to apply the ointment thinly and flatly. Additionally, symptoms of radiation dermatitis typically appear gradually a few weeks after the start of RT [8, 9, 32], highlighting the difficulty of encouraging patients to perform appropriate preventive measures from the early stages of treatment [33]. At our department, during interviews, patients are provided with instructions on the importance and methods of ointment application, using each patient’s dose distribution map of RT. This approach is intended to encourage patients to continue self-care independently as much as possible.

Moving forward, enhanced supportive care should be developed for patients with G8 scores ≤ 12 to improve their understanding and ability to maintain self-care activities. Strategies could include visual aids, such as illustrated instructional materials [34], and increasing the frequency of supportive care sessions from once per week to twice per week to provide more comprehensive assistance. Integrating the G8 score into the treatment plan may help tailor treatment approaches based on patients’ tolerance levels; for those with extremely low G8 scores and significant frailty, a shift toward irradiation focused on symptom palliation may be necessary [22, 35]. Additionally, shortening the duration of radiotherapy through hypofractionated RT could be considered to reduce the treatment burden [36, 37].

There are some limitations of our study. First, it is a single-institution, retrospective study, which may limit the generalizability of the findings. Conducting multi-center prospective studies in the future will enable us to more clearly establish the causal relationship between G8 scores and self-care activities. Second, the sample size was small, particularly in the initially non-independent group, may have influenced statistical power. Since the study focused specifically on patients ≥ 65 years, the overall cohort size was constrained. Furthermore, the number of patients who experienced self-care decline was limited (N = 15), which restricted the multivariate logistic regression model to only two variables. Although other factors such as disease stage or chemotherapy may potentially influence self-care capacity, including them in the model would have increased the risk of overfitting and reduced model reliability. Future studies with larger sample sizes are warranted to allow for more comprehensive multivariable models or stratified analyses. Third, setting the use of gauze dressing as self-care evaluation item may have introduced bias. At our facility, gauze dressing is not routinely recommended for all cases; it is typically initiated only when dermatitis becomes severe. For some patients, dermatitis remained mild throughout the treatment period, which may have led to an underestimation in this analysis. Nonetheless, the conclusion that lower G8 scores are associated with a decline in self-care remains robust.

In conclusion, the G8 screening tool serves as a practical and reliable predictor of self-care challenges during RT and CRT in patients with HNSCC. Its integration into routine clinical practice enables early identification of at-risk individuals, allowing for timely nursing interventions and tailored support strategies. By addressing the unique needs of this population, the G8 tool contributes to　better treatment outcomes, fewer complications, and an improved quality of life for elderly cancer patients.

## Supplementary Information

Below is the link to the electronic supplementary material.

ESM 1 (DOCX 36.4 KB)

## Data Availability

No datasets were generated or analysed during the current study.
