# Supplementary material for: Association between geriatric 8 (G8) scores and self-care decline in elderly patients with head and neck squamous cell carcinoma undergoing radiotherapy
Source: Support Care Cancer. 2025 Sep 16;33(10):855. doi: 10.1007/s00520-025-09936-2 (PMC12441079; doi:10.1007/s00520-025-09936-2)
Supplement: Supplementary file 1 — (DOCX 36.4 KB) [file 520_2025_9936_MOESM1_ESM.docx]

**Supplementary Materials**

| Table S1. Details of the Geriatric 8 screening tool | | |
| --- | --- | --- |
|  | Item | Score |
| 1 | Has food intake declined over the past 3 months due to loss of appetite, digestive problems, chewing or swallowing difficulties? | 0: severe decrease in food intake  1: moderate decrease in food intake  2: no decrease in food intake |
| 2 | Recent weight loss (< 3 months) | 0: weight loss > 3 kg  1: does not know  2: weight loss between 1 and 3 kg  3: no weight loss |
| 3 | Mobility | 0: bed or chair bound  1: able to get out of bed/chair but does not go out  2: goes out |
| 4 | Neuropsychological problems | 0: severe dementia or depression  1: mild dementia or depression  2: no psychological problems |
| 5 | BMI: weight in kg/ (height in m^2^) | 0: BMI < 19  1: 19 ≤ BMI < 21  2: 21 ≤ BMI < 23  3: BMI ≥ 23 |
| 6 | Do you take more than 3 medications per day? | 0: yes  1: no |
| 7 | In comparison with other people of the same age, how does the patient consider his/her health status? | 0: not so good  0.5: does not know  1: just as well  2: better |
| 8 | Age | 0: >85 years  1: 80–85 years  2: ≤80 years |
|  | Total Score | 0-17 |
|  | Abbreviation, BMI: body mass index. | |

| Table S2. The details score of the Geriatric 8 screening tool. | | | | |
| --- | --- | --- | --- | --- |
|  | Item | Score | Number of patients (*N* = 66) | Mean Score |
| 1 | Food intake in the last 3 months | 0 | 2 | 1.9 |
|  |  | 1 | 4 |  |
|  |  | 2 | 60 |  |
| 2 | Recent weight loss (< 3 months) | 0 | 10 | 2.3 |
|  |  | 1 | 1 |  |
|  |  | 2 | 13 |  |
|  |  | 3 | 42 |  |
| 3 | Mobility | 0 | 1 | 1.9 |
|  |  | 1 | 4 |  |
|  |  | 2 | 61 |  |
| 4 | Neuropsychological problems | 0 | 1 | 1.9 |
|  |  | 1 | 4 |  |
|  |  | 2 | 61 |  |
| 5 | BMI: weight in kg/ (height in m^2^) | 0 | 9 | 2.0 |
|  |  | 1 | 13 |  |
|  |  | 2 | 12 |  |
|  |  | 3 | 32 |  |
| 6 | Polypharmacy (Takes ≥3 medications per day) | 0 | 39 | 0.4 |
|  |  | 1 | 27 |  |
| 7 | Self-rated health status (compared to the same age people) | 0 | 9 | 1.3 |
|  |  | 0.5 | 6 |  |
|  |  | 1 | 18 |  |
|  |  | 2 | 33 |  |
| 8 | Age | 0 | 5 | 1.7 |
|  |  | 1 | 7 |  |
|  |  | 2 | 54 |  |
|  | Total Score |  |  | 13.5 |
| *Abbreviations*, BMI: body mass index. | | | | |

| Table S3. Age-stratified comparison of predictive performance of G8 score and PS for self-care decline. | | | | |
| --- | --- | --- | --- | --- |
| Age group | Variables | AUC | Sensitivity (%) | Specificity (%) |
| 65-73 | G8 | 0.68 | 66.7 | 54.5 |
|  | PS | 0.62 | 33.3 | 90.9 |
| ≥74 | G8 | 0.71 | 75.0 | 64.3 |
|  | PS | 0.56 | 33.3 | 78.6 |
| *Abbreviation:* G8: geriatric 8. PS: performance status. AUC: area under the curve. | | | | |
